# Dataset on factors influencing revisit intention to Jeju Island during the COVID-19 pandemic

**DOI:** 10.1016/j.dib.2025.111412

**Published:** 2025-02-19

**Authors:** Hwa-soon Lim, Mona Chang

**Affiliations:** Department of Tourism Development, Jeju National University, Jeju, 63243, Republic of Korea

**Keywords:** Demographic analysis, Social media impact, Survey data, Tourism management, Travel anxiety, Travel desire

## Abstract

This dataset explores factors influencing tourist revisit intentions to Jeju Island during the COVID-19 pandemic. Collected through an online survey of 930 participants in February 2022, the data examines seven key variables: pandemic risk perception, social media impact, destination management image, travel anxiety, travel desires, attitudes toward Jeju travel, and revisit intentions. A multi-stage probability-proportional-to-size sampling method was used, covering 16 metropolitan cities and provinces in Korea, with stratification by region, gender, and age to ensure representativeness.

The dataset includes raw survey responses, a detailed codebook, and variable descriptions, with all items measured on a 7-point Likert scale and mandatory responses to ensure completeness. Quality control measures, such as response pattern analysis and time monitoring, were implemented to maintain reliability. Publicly accessible via Mendeley Data, this dataset provides valuable insights for researchers and tourism professionals seeking to analyze tourist behavior during crises and develop effective strategies for tourism recovery and crisis management.

Specifications Table

**Table:** 

Subject	Tourism, Leisure and Hospitality Management.
Specific subject area	Tourist behavior and revisitation during COVID-19*.*
Type of data	Raw data in datasheet, Table (.csv format, processed), Supporting materials (codebook and text version of survey-summarized questions).
Data collection	The sampling framework was based on the National Travel Survey methodology established by the Ministry of Culture, Sports and Tourism of Korea (2021). A multi-stage probability-proportional-to-size systematic sampling approach was employed through a professional research company. Data collection took place in February 2022, yielding 930 valid responses from an initial panel of 14,000 potential respondents (a 6.64 % response rate). The sample represents 16 metropolitan cities and provinces across Korea (excluding Jeju Island). The sample selection process involved primary stratification by regional population distribution and secondary stratification by gender and age within each region. Additional screening criteria required respondents to have visited Jeju Island within the past year to ensure relevance to the study. The survey was conducted in Korean to ensure accurate comprehension, and all items required mandatory responses to prevent missing data. Systematic monitoring during data collection maintained proportional representation across regions, ranging from 0.6 % for Sejong to 26.6 % for Gyeonggi Province, closely aligning with national population statistics (with most differences within ±0.3 %). Quality control measures included analyzing response patterns, monitoring completion times, and conducting comprehensive data validation procedures to ensure data integrity. These checks helped to confirm the reliability of the responses and the overall quality of the dataset.
Data source location	- Location: Republic of Korea (covering 16 administrative regions: 8 metropolitan cities and 8 provinces, excluding Jeju Island) - Coordinates: 35.9078° N, 127.7669° E
Data accessibility	Repository name: Mendeley Data Data identification number: https://doi.org/10.17632/m7tbjtbx8z.5 Direct URL to data: https://data.mendeley.com/datasets/m7tbjtbx8z/5 Instructions for accessing these data: Publicly available
Related research article	H.S. Lim, M. Chang, Tourist Motivation and Revisitation in Jeju During COVID-19: Insights and Post-Pandemic Implications, Glob. Bus. Finance Rev. 29(1) (2024) 85–100. https://doi.org/10.17549/gbfr.2024.29.1.85 https://scholar.kyobobook.co.kr/file/view?downOrView=pdf&schlrCmdtcode=4010068762242&artlNum=16286800&artlName=Tourist%20Motivation%20and%20Revisitation%20in%20Jeju%20During%20COVID-19%3A%20Insights%20and%20Post-Pandemic%20Implications

## 1 Value of the Data

• This dataset provides comprehensive insights into tourists’ attitudes and their intentions to revisit Jeju Island [1]. It highlights the potential of integrating the Stimulus-Organism-Response (S-O-R) model [2] with the Theory of Reasoned Action (TRA) [3] to understand the psychological processes influencing tourist decision-making. The data structure enables researchers to test and compare these psychological theories in destination management contexts, with variables specifically designed to capture both external stimuli and internal cognitive responses.
• The dataset enables the exploration of the psychological impacts of social media and the pandemic on tourism as well as the image of destination management, including their roles in influencing travel-related fears and desires. Each measured construct serves a specific purpose in understanding tourist behavior: risk perception captures crisis response, social media impact reflects information influence, and travel anxiety/desire represents psychological states. The measurement scales align with national tourism statistics [4], allowing for comparative analysis with official tourism data while providing deeper insights into the psychological mechanisms underlying tourist decisions during crisis periods.
• Using Jeju Island as a case study, this dataset reflects broad domestic tourism trends during the COVID-19 pandemic [5]. With a large sample size collected nationwide, researchers can utilize it for demographic comparisons of tourist attitudes across different regions and social strata in Korea and for comparative analysis with other countries. The sampling methodology follows the National Travel Survey framework, ensuring robust representation for comparative analysis with other countries.

The dataset is valuable for tourism operators in developing targeted marketing strategies and crisis preparedness plans. Researchers can utilize this dataset to further analyze tourist perceptions and behaviors through various analytical approaches, from descriptive statistics to advanced modeling techniques such as SEM. It supports replicating existing studies and exploring multiple methodological approaches, providing insights into tourism dynamics within the context of external stimuli and internal cognitive responses.

## 2 Background

The survey aimed to explore the determinants and mechanisms behind tourists’ intentions to revisit Jeju Island during the COVID-19 pandemic. Relevant academic literature was reviewed to design the survey questionnaires. For instance, Kement et al. [6] provided insights into risk perception, while Matiza and Kruger [7] highlighted the impact of social media. The concept of destination management image was explored based on the work of Hassan and Soliman [8]. Travel anxiety was examined by reviewing Rather [9], and Gursoy et al. [10] provided a basis for understanding travel desire. The attitude toward travel destinations was also referred to by Kement et al. [6], and the intention to revisit for repeat tourists was further guided by Chew and Jahari [11]. Our objective was to gather data on these factors and expand our understanding of how they influence travel desires and revisit intentions. We adapted a previous research design based on the S-O-R theory measuring tourist attitudes, as seen in Isa et al. [12]. We expanded it by integrating it with the TRA theory [13] to better fit the context of Jeju Island during the pandemic. The data collected pertains to both macro and micro levels, encompassing national and regional perceptions. Key concepts covered in the survey include risk perception, social media influence, destination management image, travel anxiety/desires, and their combined effects on travel behavior. Furthermore, it examines tourists’ psychological responses to external stimuli and their impact on decision-making processes. In addition to supporting the findings of the related research article by Ren et al. [14], this dataset offers more detailed insights into the factors shaping tourist behavior. It enables further investigations and comparative analyses with other contexts.

## 3 Data Description

The dataset consists of survey responses from 930 domestic tourists who visited Jeju Island during the COVID-19 pandemic. It captures key variables related to tourists’ attitudes and revisit intentions, structured using a 7-point Likert scale framework. The dataset aligns with the National Travel Survey methodology developed by the Ministry of Culture, Sports and Tourism of Korea [4], ensuring broad representation across demographic and regional categories.

**Fig. 1:**
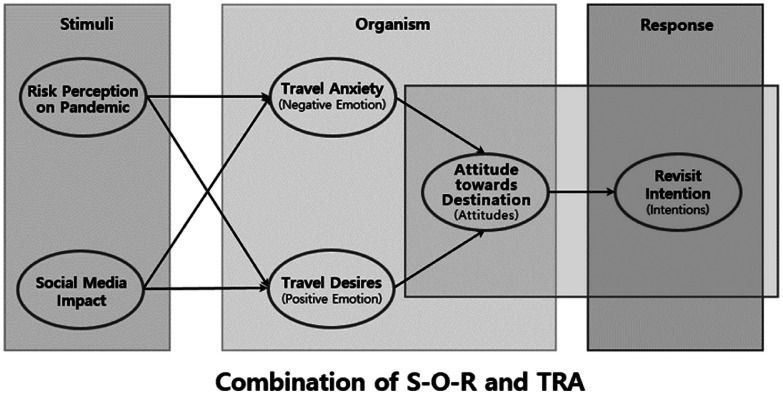
Integration of Stimulus-Organism-Response (S-O-R) and Theory of Reasoned Action (TRA) Models in the study. *Source: Authors’ own elaboration.

The dataset includes seven key variables: Risk Perception on Pandemic (RPP), Social Media Impact (SMI), Destination Management Image (DMI), Travel Anxiety (TA), Travel Desire (TD), Attitude toward Destination Traveling (ATD), and Revisit Intention (RI). Each variable was measured using multiple reflective indicators, ensuring comprehensive data coverage. The dataset was designed to facilitate the study of tourists’ psychological responses and behavioral intentions during crisis periods. Fig. 1 illustrates the theoretical framework combining the S-O-R and TRA models.

The files associated with this data-in-brief article include three main components:

(1) Survey_Summarized questions.docx: Includes detailed documentation of survey questions, covering 32 items related to tourism behavior and demographic characteristics.
(2) Revisit Intention to Jeju_930.csv: Contains raw survey data from 930 participants, with no missing responses.
(3) Codebook_Survey data.docx: Provides comprehensive descriptions of all variables, coding schemes, and calculation methods, enabling consistent and reproducible analysis.

Table 1 presents the measurement scales for the seven latent variables, each measured on a 7-point Likert scale. Note that DMI, while included in the dataset, was not applied in the original study's SEM model to avoid complexity.

**Table 1:** Latent Variables and their Measurement Scales.

Field Name	Type	Description
Latent variables 1 (5 items)	7-point Likert scale	Risk Perception on Pandemic (RPP)
Latent variables 2 (5 items)	7-point Likert scale	Social Media Impact (SMI)
Latent variables 3 (5 items)	7-point Likert scale	Destination Management Image (DMI)*
Latent variables 4 (5 items)	7-point Likert scale	Travel Anxiety (TA)
Latent variables 5 (4 items)	7-point Likert scale	Travel Desire (TD)
Latent variables 6 (4 items)	7-point Likert scale	Attitude toward Destination Traveling (ATD)
Latent variables 7 (4 items)	7-point Likert scale	Revisit Intention (RI)

⁎ Not applied in the Structural Equation Modeling (SEM) research model in the original study.

Table 2 compares our sample characteristics with national statistics, demonstrating the dataset's representativeness. The sampling followed a systematic approach aligned with national tourism statistics across multiple dimensions to enhance the validity of the results. The travel frequency distribution in our sample closely matches national patterns, with differences remaining within ±0.4 %. The demographic representation highly aligns with national statistics, particularly in gender distribution, showing only *a* ± 0.1 % difference. Age distribution across five groups (20 s through 60 s and above) demonstrates minimal variation from national statistics, with differences not exceeding ±0.3 %. The regional coverage encompasses all 16 administrative regions of Korea, with most regions showing variations within ±0.1 % of national population distributions, though Gyeonggi Province shows a slightly larger difference of −1.1 %. While the dataset also includes information on education, occupation, and income distributions as sample characteristics, direct comparisons with national statistics for these variables are unavailable due to different database classification systems.

**Table 2:** Comparison of Sample and National Population Distribution.

Variables Sample (*n* = 930) National Statistics* Difference		N	%	%	%
**T_Frequency**	*Travelling frequency to Jeju*				
	1, <3 times/year	332	35.7	36.0	−0.3
	2, 3 times/year	334	35.9	35.5	+0.4
	3, >3 times/year	264	28.4	28.5	−0.1

**Gender**	*Gender*				
	1, Male	464	49.9	49.8	+0.1
	2, Female	466	50.1	50.2	−0.1

**Age**	*Age group*				
	1, 20s	139	14.9	15.1	−0.2
	2, 30s	314	33.8	33.5	+0.3
	3, 40s	289	31.1	31.0	+0.1
	4, 50s	154	16.6	16.8	−0.2
	5, 60 s & above	34	3.7	3.6	+0.1

**Education**	*Education level*				
	1, High school	154	16.6		
	2, College	130	14.0		
	3, University	582	62.6		
	4, Graduate school	64	6.9		

**Occupation**	*Occupation*				
	1, White collar	394	42.4		
	2, Professional	77	8.3		
	3, Government Official	34	3.7		
	4, Service industry	49	5.3		
	5, Self-employed	62	6.7		
	6, Housewife	97	10.4		
	7, Student	50	5.4		
	8, Manufacturer	60	6.5		
	9, Others	107	11.5		

**Income**	*Income/month (US$)*				
	1, < $ 1000	136	14.6		
	2, $1000 ∼ $1999	86	9.2		
	3, $2000 ∼ $2999	251	27.0		
	4, $3000 ∼ $3999	186	20.0		
	5, $4000 ∼ $4999	106	11.4		
	6, ≥ $5000	165	17.7		

**Region**	*Nationwide Special/Metropolitan City, Province*				
	1, Seoul Special City	175	18.8	18.7	−0.1
	2, Busan Metropolitan City	61	6.6	6.5	−0.1
	3, Daegu Metropolitan City	43	4.6	4.7	0.1
	4, Incheon Metropolitan City	54	5.8	5.7	−0.1
	5, Gwangju Metropolitan City	26	2.8	2.9	0.1
	6, Daejeon Metropolitan City	26	2.8	2.9	0.1
	7, Ulsan Metropolitan City	21	2.3	2.2	−0.1
	8, Sejong Special City	6	0.6	0.6	0.0
	9, Geyonggi Province	248	26.6	25.6	−1.1
	10, Gangwon Province	28	3.0	2.9	−0.1
	11, Chungbuk Province	29	3.1	3.1	0.0
	12, Chungnam Province	39	4.2	4.2	0.0
	13, Jeonbuk Province	32	3.4	3.5	0.1
	14, Jeonnam Province	33	3.5	3.5	0.0
	15, Geyongbuk Province	48	5.2	5.2	0.0
	16, Geyngnam Province	60	6.5	6.5	0.0

⁎ Source: Ministry of Culture, Sports and Tourism (2021), National Travel Survey. ^†^Note: Education, occupation, and income distributions are reported for sample characteristics only, as direct comparisons with national statistics are not available due to different classification systems.

The dataset encompasses multiple key themes in tourist behavior research. First, it captures the individual characteristics of respondents, including their travel frequency and demographic profiles that align with national tourism patterns. Second, it measures tourists’ risk perceptions during the pandemic and their image of destination management practices. Third, the dataset includes measurements of social media's influence and various psychological responses related to travel, such as anxiety and desire. Finally, it contains data on behavioral intentions and decision-making factors influencing tourists’ revisit intentions to Jeju Island.

To ensure high data quality and integrity, systematic validation procedures were applied during data collection. This included response pattern analysis to detect inconsistencies, time monitoring to filter inattentive responses, and demographic stratification to ensure representativeness. All responses were complete, and no missing data were observed, enhancing the dataset's reliability for further analysis.

## 4 Experimental Design, Materials and Methods

### 4.1 Research context and rationale

This study investigates the factors influencing domestic tourists’ intentions to revisit Jeju Island amidst the COVID-19 pandemic, focusing on critical psychological and behavioral variables shaped by the ongoing health crisis. As one of South Korea's most prominent tourist destinations, Jeju Island attracts millions annually, accounting for a substantial share of domestic tourism [4]. Due to pandemic-related travel restrictions, international tourist flows have drastically declined, shifting the focus toward sustaining and revitalizing the domestic tourism market. Thus, understanding the specific factors influencing domestic revisitation intentions to Jeju is crucial to developing effective recovery strategies that adapt to changing tourist behaviors and preferences during prolonged uncertainty [15].

### 4.2 Sampling framework and data collection

The sampling framework for this study followed the National Travel Survey methodology developed by Korea's Ministry of Culture, Sports, and Tourism [4]. It employed a multi-stage probability-proportional-to-size systematic sampling approach through a professional research agency. Only individuals who visited the island within the past year were selected to ensure relevance to Jeju Island, as recent visitors are more likely to hold accurate and meaningful perceptions of the destination under pandemic conditions. The sample was stratified first by regional population to reflect Korea's demographic distribution, followed by further stratification by age and gender within each region to ensure a balanced representation. This approach allowed the study to capture a diverse yet representative group of domestic tourists with relevant and timely insights regarding their revisitation intentions toward Jeju Island. Only tourists who had visited Jeju within the past year were eligible, ensuring the dataset represented recent travel experiences and attitudes during the pandemic. Data were collected in February 2022, with a response rate of 6.64 % (930 valid responses from an initial 14,000 respondents) across Korea's 16 metropolitan cities and provinces (excluding Jeju itself).

### 4.3 Quality control in survey administration

Several rigorous quality control measures were implemented throughout the data collection process to maintain data integrity and ensure high-quality responses. First, the survey was administered in Korean to maximize comprehension accuracy among participants, with mandatory responses required for each item to eliminate missing data. Second, response pattern analysis was conducted to identify and exclude responses exhibiting uniform or straight-line answering, which can indicate inattentive or automated responses. Third, completion times were closely monitored, with responses completed in unusually short times filtered out to ensure that participants engaged thoughtfully with each question. Finally, comprehensive data validation checks were performed to verify that the demographic and geographic distributions in the collected data adhered closely to the sampling quotas and criteria established in the study design, ensuring the representativeness and reliability of the sample across all regions and demographic groups. These systematic measures collectively ensured a high-integrity dataset suitable for subsequent analysis. Variations remained within a narrow margin (mostly ±0.3 %), except for a slight underrepresentation in Gyeonggi Province (−1.1 %). Key quality control measures included response pattern analysis, completion time monitoring, and comprehensive data validation checks [16] to confirm that responses were consistent with established sampling quotas and criteria.

### 4.4 Survey design and instrument development

The survey instrument included items designed to measure the influence of the pandemic on tourist behaviors, employing seven latent variables grounded in the Stimuli-Organism-Response (S-O-R) framework and Theory of Reasoned Action (TRA). The latent variables included Risk Perception on Pandemic (RPP), Social Media Impact (SMI), Destination Management Image (DMI), Travel Anxiety (TA), Travel Desire (TD), Attitude toward Destination Traveling (ATD), and Revisit Intention (RI). Each construct was measured with multiple items (4–5 items per construct) on a 7-point Likert scale, assessing the respondents’ perceptions, emotional responses, and behavioral intentions [17].

### 4.5 Operationalization and constructs

The constructs were designed to capture key psychological and behavioral factors influencing tourists' decisions to revisit Jeju Island during the COVID-19 pandemic. Each variable was measured using multiple items on a 7-point Likert scale to ensure a comprehensive and accurate representation of tourist behavior. Risk Perception on Pandemic (RPP) was used to assess perceptions of health and safety risks [18]. Social Media Impact (SMI) assessed the influence of online content on travel decisions [19]. Travel Anxiety (TA) measured concerns related to traveling during a pandemic [20], while Travel Desire (TD) explored tourists’ aspirations to travel despite the risks [20]. Attitude toward Destination Traveling (ATD) captured overall perceptions of Jeju Island as a destination, and Revisit Intention (RI) focused on tourists’ likelihood of returning to the island in the future.

### 4.6 Data preparation and statistical checks

Data integrity was ensured through rigorous quality control measures, including response pattern analysis to identify inconsistencies, time monitoring to exclude inattentive responses, and demographic stratification to maintain representativeness. Variance Inflation Factor (VIF) values were calculated to confirm the absence of multicollinearity, with all values below the critical threshold of 5 [16]. These steps ensured that the dataset was reliable, consistent, and ready for further analysis of tourist behavior and decision-making processes.

### 4.7 Ethical considerations

Participation in the survey was entirely voluntary, and respondents were assured of anonymity and confidentiality. The research was conducted following ethical guidelines, ensuring respondents’ data was stored securely and used solely for research purposes.

## Limitations

One limitation of the dataset is the sample size. The research company employed had a large panel base, ensuring a diverse and representative sample within the budget. However, there remains a question about whether the sample size was sufficient to ensure reliability for a nationwide survey excluding Jeju Island. Since this study was not funded by external sources, budget constraints were a significant limitation.

Another limitation is that the Destination Management Image (DMI) variable was excluded from the original study's Structural Equation Modelling (SEM). Although this decision was made to prevent over-complicating the model, further analysis incorporating this variable would be necessary to understand its impact fully.

Lastly, while the survey was designed to minimize ambiguity by not including “I don't know” or “uncertain” options, this might have forced respondents to choose an option even when unsure, potentially affecting the accuracy of the responses. Despite these limitations, the dataset provides valuable insights into the factors influencing tourists’ intentions to revisit Jeju Island during the COVID-19 pandemic*.*

## Ethics Statement

We engaged a research company to conduct a nationwide online survey with approximately 40,000 panelists, resulting in 930 complete responses. Consent for personal information protection was obtained during the survey process. The Jeju National University review board exempted the study from requiring ethical approval as it involved an online survey that did not collect sensitive personal data. The authors adhered to all ethical guidelines, including the Personal Information Protection Act (PIPA) of the Republic of Korea, and secured consent from all participants under the Act.

## CRediT authorship contribution statement

**Hwa-soon Lim:** Conceptualization, Methodology, Writing – review & editing, Supervision. **Mona Chang:** Software, Validation, Writing – original draft, Writing – review & editing, Visualization, Project administration.

## Data Availability

Mendeley DataDataset on Motivation and Revisitation in Jeju for Post-Pandemic Implications (Original data).

## Appendix Supplementary materials

Image, application 1
